# The Effect of Dietary Supplementation of Vitamin E, Selenium, Zinc, Folic Acid, and N-3 Polyunsaturated Fatty Acids on Sperm Motility and Membrane Properties in Dogs

**DOI:** 10.3390/ani9020034

**Published:** 2019-01-24

**Authors:** Salvatore Alonge, Monica Melandri, Raffaella Leoci, Giovanni M. Lacalandra, Michele Caira, Giulio G. Aiudi

**Affiliations:** 1 Società Veterinaria “Il Melograno” Srl, via Cavour 48, 21018 Sesto Calende, Varese, Italy; drmonicamelandri@gmail.com; 2 Dipartimento di Medicina Veterinaria (DiMeV), Sezione di Clinica Ostetrica, University of Bari “Aldo Moro”, via per Casamassima km 3, 70010 Valenzano, Bari, Italy; leocivet@yahoo.it (R.L.); giovannimichele.lacalandra@uniba.it (G.M.L.); michele.caira@uniba.it (M.C.); giulioguido.aiudi@uniba.it (G.G.A.)

**Keywords:** canine semen, diet supplementation, dog, selenium, spermatozoa, sperm motility, vitamin E, vitamin B9, zinc

## Abstract

**Simple Summary:**

Practitioners look for specific treatments to solve sub-fertility, which nowadays represents a common challenge in canine reproduction. In human, as well as in veterinary medicine, several dietary protocols have been developed to relieve poor sperm concentration and function. They both are constraining factors of breeding programs. Particularly, food supplementation should include micronutrients and anti-oxidants in balanced amounts, exploiting their synergistic actions. Thus, the cumulative effect of vitamin E, selenium, zinc, folic acid, and n-3 polyunsaturated fatty acids (PUFA) on sperm motility and membrane properties was investigated in healthy normospermic dogs. Results of a 90-day-long supplementation period were compared to a control group, not receiving any dietary integration. The positive results obtained in healthy subjects on sperm motility and membrane properties lead us to consider the opportunity to further use the dietary strategy to improve seminal parameters in sub-fertile dogs, too, as reported for other species. Food supplementation and a balanced diet can be pointed out as cheap and safe alternatives within an innovative multimodal approach to improve reproductive performances in healthy dogs.

**Abstract:**

Sub-fertility represents a common challenge in canine reproduction. Different protocols, supplementing daily given quantities of micronutrients, were investigated to improve poor sperm concentration and/or function, which represent breeding major constraining factors in the canine species. Little information is available for dogs concerning the effect of a daily supplementation with a complex of vitamin E, zinc, selenium, folic acid, and n-3 polyunsaturated fatty acids (PUFA) on semen quality. Thus, the present study investigated this effect on semen motility and sperm membrane properties. Serial semen analyses from fourteen healthy normospermic dogs, fed with the same commercial diet, were performed on Days 0 (T0), 30 (T30), 60 (T60), and 90 (T90). Seven dogs were randomly included in the treatment (T) group, receiving a supplementation of vitamin E, zinc, selenium, folic acid, n-3 PUFA; and seven other subjects composed the control (C) group. Total Sperm Count (TSC), Computer-Assisted Sperm Analysis (CASA) indexes, mortality, and functional membrane integrity were assessed. The ANOVA compared results between groups and sampling times (*p* < 0.05). From T60, the supplementation significantly improved TSC, progressive motility, functional membrane integrity, and decreased mortality. Present results lead us to consider ameliorative effects of a two-month healthy diet supplementation on canine spermatozoa. The positive effects of the described balanced integration of micronutrients on sperm motility and prevention of oxidative stress should be considered, especially when decreased seminal parameters may result from inadequate intake, reduced absorption, increased losses or demand, or to attenuate the impact of age.

## 1. Introduction

Currently, the reproductive failure in the canine species is one of the main aspects involving breeders and practitioners, who look for specific treatments to face it. Male factor is responsible for many cases of suspected sub- and infertility, especially when presenting with an altered semen analysis. Mainly, some male reproductive failures seem to result from definite nutritional deficiencies or adverse environmental and lifestyle factors [1,2]. According to the literature, the major constraining factors of breeding programs are represented by poor sperm concentration and/or function. Thus, several authors investigated different protocols to improve semen quality, in both human and veterinary medicine, supplementing a certain daily intake of micronutrients [3,4,5,6]. Little information is available for dogs [7,8].

Several human and animal clinical research studies suggest that, among nutritional factors, fish-derived n-3 polyunsaturated fatty acids (PUFA) can exert a positive effect on sperm motility and fertility; since they cannot be synthesized de novo by animals, they need to be provided with the diet [3,4,5,6,9]. Moreover, some authors suggested that n-3 PUFA-enriched diets manage to change the n-6:n-3 ratio of spermatozoa, improving sperm motility [9]. These results would imply that dietary n-3 PUFA might help improve sperm functions, especially motility, by modifying membrane properties, as well as decreasing spermatozoa lipidic peroxidation [10]. On the other hand, it was recently demonstrated that dietary n-3 PUFA modify the spermatozoa fatty acid (FA) profile [9,11] and promote the susceptibility of membrane PUFA to lipidic peroxidation [12,13], thus change membrane integrity and function, and subsequently modify sperm motility and fertility [13,14].

Other micro-elements [1,2,15,16,17] act directly or indirectly on sperm metabolism. Different authors managed to demonstrate that nutrient deficiencies can lead to impaired sperm quality, through defective spermatogenesis, or by generating intense body oxidative stress [18]. Oxidative alterations provoke sperm dysfunctions, such as loss of motility and viability, and impairment of sperm–oocyte fusion [19]. Thus, antioxidants can play a crucial role in protecting male germ cells against oxidative damage [20], preventing the loss of motility and the decreased capacity for sperm–oocyte fusion [19,21].

Vitamin E (Vit E) is one of the major antioxidants in the body and plays a major role in protecting different organs against oxidative stress and stabilizing the sperm membranes by complex formation [22,23]. This vitamin protects the testis from oxidative damage and improves sperm quality in goats [24], sheep [25], and rabbits [26,27].

Furthermore, several trace elements act in the male reproductive process thanks to their high activity at molecular level. They are present in the body at very low concentrations. Among them, the availability of Zinc (Zn) and Selenium (Se) plays a beneficial role in the organism, as they influence the corresponding enzymes activity, which carries sperm protection against oxidative damage: superoxide dismutase (SOD), and glutathione peroxidase (GPx). The metal ion zinc is a co-factor of SOD, while selenium is a co-factor of GPx [28,29].

In body tissues, zinc represents the second most abundant element after iron. In reproduction, zinc has numerous important functions and is essential for conception, implantation, and a favorable pregnancy outcome [30,31]. Zinc is available in high concentrations in the seminal fluid and could play a multifaceted role in sperm functional properties, influencing the fluidity of lipids, thus in the stability of biological membranes [32], sperm chromatin integrity [33], free oxygen radical production [34], and capacitation and acrosome reaction processes [35].

Owing to its role as a cofactor of the glutathione peroxidase [36,37], the antioxidant performance of selenium was investigated in different clinical studies, in boars, cockerels, ganders, gilts, rabbits, rats, and dogs. The possible synergic effect of selenium and vitamin E supplementation on semen quality was elucidated for several species [7,8,12,26,38,39,40,41,42].

Finally, in human medicine, vitamin B9 (Vit B9), or folic acid, has always been considered fundamental in pregnancy, because it tends to protect and promote the embryonic development. vitamin B9 is also essential for the synthesis of proteins and DNA [43]. The importance of its supplementation was recently highlighted for the canine species, too, especially in the bitch [44]. This water-soluble vitamin cannot be stored in the body, thus must be regularly taken with food. As for other micronutrients, some studies were carried on also with folic acid to investigate its role in the male reproductive function; its supplementation turned out to be associated with an excellent quality of the sperm, increasing both concentration and count in men [45,46].

Thus, according to literature studies, performed in either normospermic or presumptively lowered fertility patients, a proper micronutrients and antioxidants supplementation could improve sperm quality through the alleviation of nutrient deficiencies as well as the reduction of oxidative stress [7,8,47,48].

Furthermore, to the authors’ knowledge, no data are available for dogs, concerning the effect of a daily supplementation with a complex of vitamin E, zinc, selenium, folic acid, and n-3 PUFA on semen quality. Thus, the present study investigated this effect on semen motility and sperm membrane properties in healthy normospermic dogs.

## 2. Materials and Methods

### 2.1. Animals

Patients were selected for the study among those presented at the University Hospital as usual semen donors for artificial insemination. Patients were required at least two monthly semen collections in the two months immediately before the beginning of the study, with normospermic results, to avoid the bad effect of abstinence. Sixteen dogs of different breeds (bodyweight: 8–35 kg, age: 1.5–5 years) were simultaneously selected, to avoid any possible seasonal effect on sperm parameters. All subjects lived indoors, in very similar environmental conditions, at the owners’ place, without any remarkable difference in the respective families’ lifestyle. They were fed with dosed commercial food and water ad libitum. Each dog underwent a clinical examination, including a thorough history evaluation, as well as a male breeding soundness exam (with clinical and ultrasonographic evaluation of the reproductive organs) [49,50,51,52,53,54]. All dogs were healthy. Sexual rest was required for the whole duration of the study. One week before enrollment, it was verified that all subjects reacted positively to semen collection by manual stimulation and that they were still normospermic.

In 10 days, the diet of all dogs was gradually replaced by the same commercial dry food (Sìland, Aurora®, Milan, Italy). In the following 60 days, dogs were left to completely adapt to the new feed. The commercial food used in the study has a metabolizable energy of 3900 kcal/kg. In its analytical composition, the manufacturer reports: vitamin E 250 mg/kg, vitamin B9 1.5 mg/kg, zinc 180 mg/kg, selenium 0.27 mg/kg and n-3 PUFA 0.5%. According to the European Pet Food Industry Federation (FEDIAF) 2016 Guidelines, the total daily food intake was calculated, based on maintenance energy requirements (MER, kcal/die). The MER was calculated for each dog, considering the 4 *k* coefficients, which are represented by breed, attitude, physiologic condition, and health [55]. The daily micronutrients intake by food was as follows:

- Vitamin E (3 mg/kg body weight (BW));
- Zinc (2.4 mg/kg BW);
- Selenium (0.003 mg/kg BW);
- Folic Acid (0.02 mg/kg BW).

Since inclusion criteria required to enroll healthy animals only, two subjects were excluded from the study during the 60-day-period of adaptation to the new feed because of accidents requiring the use of non-steroid anti-inflammatory drugs (NSAIDs) and antibiotic therapy. Therefore, fourteen dogs (BW: 25–35 kg, age: 1.5–4.5 years) were finally enrolled to the experimental phase of the study, beginning with Day 0 (T0) (Table 1). Dogs were randomly assigned to the control (C) or treatment group (T), seven dogs in each group, by an automatic tool for the generation of casual numbers (based on Microsoft Office Excel). Starting from T0, the diet of the subjects enrolled in the T group was enriched with galenic food supplementation (Eureka Vet Service snc, Paruzzaro, Italy), in tablets to be added to the above-mentioned diet during the 90 days of the study, while dogs in the C group went on receiving only the cited commercial dry food.

The number of patients enrolled in the study (14) was expected to provide satisfactory statistical examination. In fact, other authors, previously working on this topic in the canine species, already obtained statistically significant results, even with fewer dogs: Risso and colleagues [48] enrolled 5 males, Kirchhoff and colleagues [8] worked on 3 patients, while Kawakami and colleagues [47] evaluated 4 dogs.

It should also be pointed out that the present study was performed on healthy normospermic dogs, to evaluate the possible effects of a dietary supplementation on seminal parameters, and to exclude any potential side effect of this complex at the given dosages on semen quality, in order to later transpose present results to non-normospermic dogs. Several studies from the literature describe similar protocols, even if with different procedures and alimentary additions, not only in the canine species [8,48], but also in rams [10] and in men [56].

### 2.2. Food Supplementation

To increase fertility, as suggested by the literature, in both human and veterinary medicine, the cumulative effect of specific trace elements and vitamins has to be considered [7,8,10,18,48,56]. In previous studies, many authors, who focused attention on the comparison between mono- and complex-integrations, finally vividly recommended the use of combined formulations to improve sperm concentration and motility [7,8,10,18,48,56]. Moreover, some authors already demonstrated that, when micronutrients can act synergistically with each other, their effect has to be considered as the enhanced sum of the partly augmentative effects of each other [18]. Thus, in light of the most recent literature, in this study, the supplementation of a complex of micronutrients was considered to expand knowledge on the effect of a food complex supplementation on changes in motility and membrane integrity of canine spermatozoa. Thus, the administered tablets were formulated as follows: vitamin E (5 mg/kg BW); zinc (3 mg/kg BW); selenium (0.007 mg/kg BW); and folic acid (0.625 mg/kg BW). Refined fish oil (25% DHA (Docosahexaenoic acid) and 10% EPA (Eicosapentaenoic acid)) was used to supplement n-3 PUFA in the tablets.

The food supplementation was provided daily during meals to the subjects in the T group (easily divisible tablets, one each 20 kg of bodyweight), throughout the 90-day-treatment period of the study.

### 2.3. Experimental Protocol

At T0, all dogs were subjected to semen sampling to evaluate seminal quantitative and qualitative parameters, the percentage of live and dead spermatozoa, and the functional integrity of the cytoplasmic membrane. This procedure allowed assessing basal seminal values after all dogs had received the same feed for two months, to avoid possible feed bias on the semen analysis. Such period is of benefit to this study, as it corresponds to the critical time required to complete an entire spermatogenesis cycle in the dog [57].

Monthly assessments (T30, T60, and T90) of semen analysis were repeated, to test the effects of diet and food supplementation on seminal parameters, compared to T0 in all dogs.

Semen was collected by digital manipulation, allowing dogs to sniff swabs of bitches in estrous, exploiting the presence of their natural estral pheromones, and collected in Falcon tubes in three separated fractions: urethral, spermatic, and prostatic.

### 2.4. Sperm Analysis

#### 2.4.1. Computer Assisted Sperm Analysis

The second seminal fraction was readily analyzed for concentration and motility using CASA (Computer Assisted Sperm Analyzer, IVOS-Sperm CASA system, Version 12.3, Hamilton Thorne, MA, USA).

Following the manufacturer’s instructions, for each analysis, a 10 µL drop from each semen sample was diluted 5 times in Tris-Fructose extender and put on Leja slide 4 chambers 20 μm (Leja Products B.V. Nieuw Vennep, The Netherlands). The Leja slide was positioned on the dedicated chamber of the microscope allowing settling before analysis. The computerized analyzer scanned five random non-consecutive microscopic fields.

The following parameters were evaluated: total number of counted cells (TSC); semen concentration; total motility, and percentage of motile spermatozoa (progressive motility). Velocity average pathway (VAP) was elaborated by the software as average velocity of smoothed cell path, expressed in µm/s. Then, the overall sperm population was divided into 4 groups, based on the velocity, according to low VAP cut-off (LVV) and medium VAP cut-off (MVV). Thus, sperms were classified as follows: rapid spermatozoa, with VAP > MVV; medium spermatozoa, with LVV < VAP < MVV; slow spermatozoa, with VAP < LVV; and static spermatozoa, represented by the fraction of those cells not moving during the analysis [58].

#### 2.4.2. Structural Membrane Integrity

As previously reported by other authors in similar clinical studies [7,10,48], the structural membrane integrity was evaluated by Eosin/Nigrosin staining method. The proportion of spermatozoa with intact cytoplasmic membrane was estimated using Eosine/Nigrosine staining, with the observation of at least 100 spermatozoa at ×400 magnification. Dead spermatozoa and those with structural alterations of the membrane appeared with a red or an intense pink head, while live sperms showed a pale white head. Dead spermatozoa, which possess an altered membrane with perforations and loss of integrity, are permeable to the dye and, therefore, appear colored after the vitality test. On the other hand, live spermatozoa are not colored, as they have an intact membrane, which prevents the dye from entering [59].

#### 2.4.3. Functional Membrane Integrity

The functional integrity of the cytoplasmic membrane of spermatozoa was evaluated by the hypo-osmotic swelling test (HOS). Therefore, spermatozoa were diluted with a hypo-osmotic solution, which entered the cells. The test was carried out adding 25 µL of semen to 250 µL of hypo-osmotic solution (25 mMol of sodium citrate dihydrate and 75 mMol D-fructose) at 37 °C, keeping this mixture in an incubator for thirty minutes. Then, 10 µL were taken and placed on a slide for objects, covered with a covering-object slide, then observed under an optical microscope (×400), counting 100 cells, which were evaluated for coiled tails, in at least five different fields. A live spermatozoon with intact cytoplasmic membrane englobes or inflates its tail due to the entrance of water: spermatozoa with intact cell membrane appear with various types of swelling of the tail, differently from those with damaged cell membrane, which, on the contrary, show no signs of swelling [60]. Sperm morphology was evaluated and the number of cells with coiled tail was defined to be subtracted from those tails that coiled after incubation.

### 2.5. Statistical Analysis

Semen analysis results at T0 were considered as basal levels for each dog, as the seminal performance at T0 was estimated to be affected only by the assumption of the same commercial diet for an entire cycle of spermatogenesis.

All collected data were reported on Excel 2010 Office files, mean ± SD values were calculated for each parameter, then statistically evaluated by ANOVA test. Data were statistically compared first within each group on each sampling time: in group T, to assess the effect of diet supplementation over time, and, in group C, to exclude the confounding effect of repeated samplings and of quality commercial diet on seminal parameters over time. Then data were statistically compared between groups on each sampling time, to verify the effect of the supplementation in group T on seminal parameters compared to controls. Results were considered significant for *p* ≤ 0.05. The statistical analysis was performed with the online tool VassarStats: Website for Statistical Computation (http://vassarstats.net, Vassar College, New York, NY, USA).

### 2.6. Ethical Guidelines Committee

The present study was performed in accordance with the ethical guidelines of the animal welfare committee. Institutional Review Board approval of the study was obtained by the University of Bari “Aldo Moro”, Ethic Committee DETO, Italy (Protocol No. 38/17 DETO; 26 June 2017). Procedures with animals were performed following good veterinary practice for animal welfare according to national laws in force (D.Lgs 116/92). Informed owner consent was obtained.

## 3. Results

No patients were lost at periodic procedures until T90.

No dogs showed difficulties related to feeding changes or supplementation use. They liked the new feed and were not reluctant to take the tablets, which were considered acceptable for all dogs enrolled.

At T0, no differences were reported for seminal parameters between groups.

The volume of the sperm-rich fraction did not statistically differ among different days of sperm collection, neither between groups nor over the duration of the study. 

Within the control group, a significant decrease in the percentage of slow-movement sperms was observed from T60, and a significant decrease in static spermatozoa was reported at T90. The supplementation significantly improved quantitative and qualitative parameters in the T group, as reported in Table 2. In the treated group, total sperm count and concentration significantly increased at T60 and T90 vs. T0, resulting also statistically higher than in the control group at T60 and T90, respectively. A similar trend was obtained also for progressive motility; an increase in rapid and medium motile sperms was observed from T60 and T90, respectively, the first resulting statistically higher than in the C group from T30 and the second from T60. Slowly motile sperms decreased at T60 and T90 vs. T0, resulting also statistically lower than in the control group at T30, T60 and T90. A significant decrease in the static sperm percentage was observed from T30 in dogs of the T group and, comparing results by time between groups, they resulted also statistically lower than in the control group from T30. Moreover, this ameliorative result of the supplementation diet was confirmed by a significant increase in sperm vitality and functional membrane integrity in the T group from T60 vs. T0, resulting also statistically better than in the control group from T30. 

## 4. Discussion

Canine sub- and infertility are common challenges in the reproduction of small animals. Breeders often require specific treatments to solve this burdensome problem. Several authors suggested different protocols to improve sperm quality [8,48].

The sperm-rich fraction volume remained constant in dogs from both groups all over the duration of the study. Our result is in agreement with literature reports [7,48]. This probably depends on the training of dogs to manual semen collection, a feature equally common to all enrolled patients, regardless of study group belonging, diet or specific supplementations.

Results obtained in the present study demonstrate that the integration of a healthy diet, enriched with a complex of vitamin E, selenium, zinc, folic acid, and n-3 polyunsaturated fatty acids, can significantly increase the number of spermatozoa, and improve motility and membrane properties of the ejaculate in healthy normospermic dogs. Similar results were reported in 15 lowered fertility dogs using selenium and vitamin E [7], in four poor quality semen dogs by vitamin E [47], and in five healthy dogs by n-3 PUFA [48]. Conversely, Kirchhoff and colleagues, in 2017 [8], failed to identify a clear trend in the seminal parameters of three healthy Cairn Terriers supplemented with vitamin E and selenium. The question on supplemented doses is still debated in the literature. Kirchhoff and colleagues (2017) [8] used vitamin E and selenium in dosages and ratios much higher than in the other mentioned studies and also than in the present report. It should be marked that, even if selenium and vitamin E are useful supplements for semen quality, their excess can damage spermatozoa [7]. It was in fact demonstrated that any excess in selenium can decrease sperm motility in men [61], while 20-fold doses of vitamin E can provoke harmful effects on spermatozoa in cocks [62].

In the present study, benefits of the enriched diet on sperm motility and membrane properties were generally statistically evident starting from a 60-day-supplementation, which approximately corresponds to the physiological length of the total duration of spermatogenesis in dogs, reported as 61.9 ± 0.14 days [57].

Present results suggest that repeated samplings do not substantially ameliorate quantitative and qualitative seminal parameters. However, repeated serial expulsions of seminal material lead to the availability of fresher sperms, preventing negative effects of their ageing along with epididymal storage [56,63], as highlighted by the statistically lower percentage of slowly moving and static sperms in the C group, since T60 and T90, respectively. On the other hand, in the T group, diet supplementation ameliorated seminal quality, resulting in an earlier decrease of the percentage of static sperms, already statistically significant at T30 vs. T0. This result is in accordance with a previous report, in which authors supplementing vitamin E and selenium, only, indicated a statistical amelioration concerning static spermatozoa at the same timing, T30 [7]. In this case, the benefits of diet supplementation do not require a whole spermatogenesis cycle to become evident because provided antioxidants prevent the negative effect of ageing on those sperm cells already present at T0, through their action against lipidic peroxidation [64]. Reports from human medicine show similar effects on sperm motility by supplementing folic acid [65] and zinc [66], starting from 90 days after the beginning of the enriched diet. However, since the reduction in static sperms percentage highlighted in the present study results more conspicuous than previously reported [7] and takes place earlier than indicated in other studies [65,66], it can be suggested that the complex supplemented in the present trial obtains benefits from the synergic actions of all its components, not only vitamin E and selenium, but also folic acid, zinc, and n-3 PUFA.

Even if it was not the purpose of the study, it was noted that all dogs enrolled in the T group presented a better quality of hair, appearing more polished.

### 4.1. Concentration and Motility

Results of the present study confirm that, as already observed in other species, an adequate dietary supplementation can improve total sperm count and semen concentration in dogs, too, as reported in this trial at T60 in the treated group.

Particularly, the synergic effects of both folic acid and zinc, compounds of the supplementation given in group T, lead to sperm production and maturation promotion, as reported by previous authors [67], with a significant increase in total sperm count and concentration.

Following the dietary supplementation, thanks to the possibilities offered by the computed sperm analysis of the sperm movement, the latter significantly increased in all dogs of the treated group. Total motility remained constant throughout the study in both groups, and within normality ranges, as no dogs were presented with lowered fertility at the beginning of the trial. However, from T60, the treated group generally obtained considerable benefits from the supplementation in terms of motility, as in this group the progressive motility and the percentage of rapid-movement sperms significantly increased, while the percentage of slow and static sperms significantly decreased. These variations are linked to the composition of the supplementation given, rich in zinc, n-3 PUFA, folic acid, and vitamin E. Sperms of all four velocity categories resulted statistically different from the respective time in the C group (higher for rapid and medium velocity, lower for static and slow velocity spermatozoa). In fact, as reported in the literature, sperm motility, one of the male fertility limiting factors, resulting from a multi-step process [68,69], is significantly influenced by zinc, which has to be considered as one of the major dietary factors affecting sperm motility [70,71,72,73,74]. Zinc levels in seminal plasma were positively associated with sperm motility [75,76]. Moreover, among other dietary factors, several experimental data from human and animal studies, indicate a positive effect of n-3 polyunsaturated fatty acids, derived from fish oil, on sperm motility and fertility [3,4,6,9]. Finally, a diet rich in folic acid and vitamin E was demonstrated to improve motility, even in the ejaculate of patients with different pathologies [18,77].

In human medicine, it is known that, compared to the normal diet, the intake of supplementations and a greater assumption of antioxidants are associated with a higher number of spermatozoa and better motility, even in healthy men of different ages [45]. Moreover, the antioxidant intake might partially mitigate the impact of age on sperm motility [64]. The same phenomenon was demonstrated also in healthy dogs by the results of the present study: although all patients enrolled were healthy and showed no signs of lowered fertility, group T dogs got statistically significant higher results of total sperm count, concentration, and progressive motility, compared to C group dogs, thanks to the specific supplementation given. A similar effect on total sperm count was obtained by Risso and colleagues (2016) [48], who only supplemented n-3 PUFA. However, the present results on progressive motility are much more marked, thanks to the synergic interaction among all integrated molecules. The n-3 PUFA supplementation is a key element to accelerate spermatogenesis, playing a key role in the development of functional sperms [48]. On the other hand, the lack of additional antioxidants could lead to minor ameliorations in terms of progressive motility. Normal spermatic metabolism leads to a great energy consumption and, under this condition, sperm cells integrated with n-3 PUFA are more prone to suffer from lipidic peroxidation, thus require a greater availability of antioxidants. Antioxidants should therefore be given with the diet supplementation, to preserve and increase progressive motility, as highlighted by the results of the present study.

In light of research reported in the literature on this topic, results of the present study, in terms of concentration and motility, can be ascribed to the cumulative effect of several compounds included in the supplementation given: vitamin E, zinc, folic acid, and n-3 PUFA.

### 4.2. Structural and Functional Membrane Integrity

Results obtained in the hypo-osmotic swelling test demonstrate the effectiveness of food supplementation in improving the quality of spermatozoa. A healthy diet, with the use of supplements, can therefore be an economical and safe way to improve quality and fertility of sperms.

Previous studies suggested that n-3 PUFA increase the incorporation of DHA in the head and in the main portion of the sperm tail, and that DHA plays a structural role, guaranteeing better plasticity and stability of the sperm membrane, resulting protective against the hypo-osmotic solution [78]. These beneficial effects could be ascribed to: the improvement of the functional integrity of the membrane, as already demonstrated in rabbits [41,79] and rams [80]; the integrity of the structural membrane of spermatozoa, as described in boars [81]; and the greater fluidity of the cytoplasmic membrane, as reported in mice [82].

According to the literature, changes in the structure of the cytoplasmic membrane and its functional activity represent essential prerequisites for the acquisition of effective motility, fertilization capacity, and embryonic development [83,84]. Changes in the fatty acid profile of the sperm membrane and its lipidic peroxidation are closely associated with variations in the physical and functional properties of the sperm cell membrane [85,86]. The supplementation with n-3 PUFA, decreasing the n-6:n-3 ratio, as already demonstrated in pigs, improves the characteristics of the cytoplasmic membrane, influencing the composition of fatty acids in spermatozoa [10]. On the other hand, the enrichment of the diet with n-3- PUFA, only, could result in spermatozoa that are more likely subject to lipidic peroxidation, which negatively affects structural and functional properties of membrane and sperm motility [13,81]. Owing to their high content in polyunsaturated fatty acids, spermatozoa are particularly sensitive to oxidative damage [87], and its prevention is considered crucial to keep normal fertility, as it can be negatively affected by high levels of reactive oxygen species (ROS) [19,21]. For this reason, it is necessary to enrich the diet with adequate levels of antioxidants, too, in order to protect male germ cells against oxidative damage [20].

In this perspective, vitamin E must always be included in the diet to reverse the negative impact of lipidic peroxidation of sperm, linked to dietary supplementation with n-3 PUFA [4,14], in order to capture free radicals and stabilize the membrane of spermatozoa [23]. Selenium, the cofactor of glutathione peroxidase, can enhance the antioxidant activity of vitamin E [36]. folic acid and zinc, too, have positively been associated with sperm quality: an adequate amount of zinc in the seminal plasma, balancing the possible excessive quantity of superoxide anions, can exert a further protective effect, thanks to its antioxidant activity [34]. Previous authors showed that zinc in seminal plasma stabilizes the cell membrane of spermatozoa [88], influencing the fluidity of lipids of biological membranes [32].

The statistically significant improvement obtained for both, structural membrane integrity and HOS values, from T60 may be ascribed to a consequence of an adequate supplementation. The n-3 PUFA positively contribute to the biochemical composition of the spermatic cytoplasmic membrane, providing better plasticity and stability, while vitamin E, selenium, zinc, and folic acid prevent its lipidic peroxidation and neutralize the possible production of reactive oxygen species. Moreover, as previously mentioned, considering the dramatically decreased percentage of static spermatozoa in the T group vs. C group, also in this case, the benefits of food supplementation can become evident even before a whole spermatogenesis cycle is completed. It might act mitigating partially the negative impact of ageing on those sperm cells already present at T0, and becoming even more evident as the duration of supplementation increases. These trace elements play a synergic role on the structural and functional membrane integrity of spermatozoa, thus on their vitality, leading to statistically significant differences reported in the results of the present study between group T and C, when comparing data for the respective times, from T30 and over.

Finally, as suggested in the literature, results of the present study confirm the importance of a balanced integration of micronutrients, avoiding any over-supplementation, which could have the undesired opposite effect. Thus, a complete excellent management of the diet, considering both the composition and the dosage of different micronutrients given with feed and specific supplementations, has to be suggested. Furthermore, since some sperm abnormalities are more related to testicular spermatogenesis, and others with epididymal maturation, further dedicated investigations would be advisable to deepen the specific site of action of the feed supplementation, analyzing the effect of this dietary integration on sperm morphology.

## 5. Conclusions

The decrease in concentration, motility, and function of canine semen could derive from inadequate food intake, reduced absorption, increased losses or augmented demand for micro-elements. Feed supplementation might mitigate the serious negative zootechnical and economic impact of sub- and infertility in canine breeding. A healthy diet, properly supplemented with vitamin E, zinc, selenium, folic acid, and n-3 PUFA for at least two months, can represent a cheap and safe way to improve quality and fertility of canine semen. As previously stated, the supplementation of breeding males should be done in a balanced and not overdone manner, underlining that over-supplementation could have undesired opposite effects. Results of the present study, according to the most recent literature, encourage considering also the alimentary approach by balanced food supplementation for the management of hypo-fertility in dogs. The ameliorating effects obtained on seminal parameters in healthy dogs lead to consider the opportunity to plan further studies to evaluate the use of the dietary strategy, when necessary, to improve seminal parameters also in subfertile subjects, as reported for other species. In the light of present results, a diet enriched with vitamin E, zinc, selenium, folic acid, and n-3 PUFA for at least two months, in suitable amounts, improves sperm quantity and quality, especially sperm count and motility, modifying physical and functional properties of the sperm cell membrane. The cumulative effect of these simultaneously integrated micronutrients has to be considered as sum of the partly ameliorative and augmentative effects of each component. The benefit of the enriched diet in ameliorating the seminal evaluation can represent an important step to optimize this peculiar feature of the male factor to the topic of fertility. The final success requires a complete careful evaluation of all the different aspects involved in the field of breeding and fertility.

## Figures and Tables

**Table 1:** Animals enrolled in the treatment (T) and control (C) group and their metabolic requirements.

Breed	Bodyweight (kg)	Age (years)	Maintenance Energy Requirement (kcal/die)	Required Daily Food Intake (kg)	Group
Amstaff Terrier	34.0	4	1703.70	0.44	T
Boxer	29.5	3	1392.38	0.36	T
Boxer	33.0	2	1514.53	0.39	T
Husky	26.0	4	1114.56	0.29	T
Golden Retriever	33.0	1.5	1499.38	0.38	T
Weimaraner	32.0	4	1627.98	0.42	T
Irish Setter	32.0	1.5	1775.98	0.45	T
German Shepherd	35.0	2	1582.90	0.41	C
Labrador Retriever	30.0	3.5	1127.28	0.29	C
English Setter	25.0	2.5	1229.80	0.32	C
Husky	27.0	4.5	937.73	0.24	C
Chow Chow	33.0	1.5	1454.11	0.37	C
Golden Retriever	34.0	2	1533.31	0.39	C
Bull Terrier	29.0	3	1375.00	0.35	C

**Table 2:** Quantitative and qualitative parameters along time, reported as mean ± SD, obtained from all dogs from the same group (Treatment and Control group). Different superscripts denote statistically significant differences between columns within groups (lowercase (a and b) to underline statistical differences within T group, uppercase (A and B) within C group); different symbols denote statistically significant differences between times between groups (# in T group vs. § in C group). HOS: hypo-osmotic swelling test.

Group	Treatment Group				Control Group			
Parameter	T0	T30	T60	T90	T0	T30	T60	T90
**Sperm-Rich Fraction Volume (mL)**	0.99 ± 0.04	1.23 ± 0.39	1.03 ± 0.10	1.07 ± 0.19	0.95 ± 0.08	1.24 ± 0.45	1.43 ± 0.73	1.14 ± 0.35
**Concentration (spz × 10^6^/mL)**	390.86 ^a^ ± 82.13	464.57 ^a,b^ ± 153.12	873.57 ^b,#^ ± 262.04	899.43 ^b,#^ ± 150.67	377.56 ± 72.48	420.38 ± 67.8	376.79 ^§^ ± 62.22	387.89 ^§^ ± 89.77
**Total Sperm Count (spz × 10^6^)**	386.57 ^a^ ± 88.22	613.71 ^a,b^ ± 392.29	892.13 ^b,#^ ± 247.01	956.00 ^b,#^ ± 175.85	358.83 ± 72.70	533.30 ± 177.53	567.36 ^§^ ± 363.18	440.6 ^§^ ± 150.8
**Total Motility (%)**	93.00 ± 7.44	96.43 ± 6.40	98.86 ± 1.07	98.71 ± 0.76	94.43 ± 6.97	95.57 ± 6.16	95.86 ± 5.28	96.29 ± 5.36
**Progressive Motility (%)**	51.29 ^a^ ± 22.51	71.86 ^a,b^ ± 9.92	77.86 ^b,#^ ± 11.51	82.86 ^b,#^ ± 7.99	55.43 ± 14.02	65.57 ± 15.49	62.14 ^§^ ± 12.05	68.43 ^§^ ± 13.59
**Rapid (%)**	73.29 ^a^ ± 10.24	83.40 ^a,b,#^ ± 6.20	86.43 ^b,#^ ± 3.64	86.79 ^b,#^ ± 3.58	71.43 ± 11.32	73.86 ^§^ ± 4.79	79.71 ^§^ ± 6.43	80.43 ^§^ ± 5.21
**Medium (%)**	6.14 ^a^ ± 3.34	8.86 ^a,b^ ± 6.54	10.86 ^a,b,#^ ± 3.08	11.00 ^b^ ± 2.31	6.43 ± 7.13	5.29 ± 3.99	5.29 ^§^ ± 6.47	8.14 ± 3.56
**Slow (%)**	11.14 ^a^ ± 5.76	5.29 ^a,b,#^ ± 2.75	2.14 ^b,#^ ± 1.68	1.79 ^b,#^ ± 1.73	12 ^A^ ± 4.69	9.86 ^A,§^ ± 3.14	6.14 ^B,§^ ± 2.95	4.71 ^B,§^ ± 2.05
**Static (%)**	9.43 ^a^ ± 3.51	2.43 ^b,#^ ± 1.62	0.86 ^b,#^ ± 0.38	0.71 ^b,#^ ± 0.49	10.14 ^A^ ± 2.1	11 ^A,§^ ± 2.56	8.86 ^A,B,§^ ± 3.64	6.71 ^B,§^ ± 3.41
**HOS (% curled)**	91.14 ^a^ ± 4.22	94.71 ^a,b,#^ ± 4.61	97.43 ^b,#^ ± 1.62	96.29 ^b,#^ ± 2.36	91.14 ± 2.95	90.57 ^§^ ± 4.27	92.14 ^§^ ± 5.51	92.71 ^§^ ± 2.25
**Sperm Viability (Eo-Nig) (%)**	91.29 ^a^ ± 5.94	94.86 ^a,b,#^ ± 3.85	98.71 ^b,#^ ± 1.25	98.71 ^b,#^ ± 0.95	91.13 ± 0.69	91.57 ^§^ ± 3.64	91.86 ^§^ ± 3.34	93.65 ^§^ ± 3.80
